# All-optical control of three-photon spectra and time asymmetry in a strongly coupled cavity polariton system

**DOI:** 10.1038/srep22560

**Published:** 2016-03-03

**Authors:** X. Zhang, R. Li, Haibin Wu

**Affiliations:** 1 State Key Laboratory of Precision Spectroscopy, Department of Physics, East China Normal University, Shanghai 200062, China

## Abstract

Manipulating the nature of photons emission is one of the basic tasks in quantum optics and photonics. The ever growing list of quantum applications requires a robust means of controlling the strongly coupled coherent interaction of photons and matter. Here, we investigate three-photon transmission spectra in a strongly coupled cavity polariton system and show that the correlation functions and transmitted photon stream can be optically manipulated. The dynamics of single photons and photon pairs at the polariton resonances can be changed by light from a single external coupling laser. At the “dark-state polariton,” three-photon transmission is a perfectly coherent field in contrast to the strong photon-bunching behavior of a typical cavity quantum electrodynamics system. When the detuned probe light is tuned to the “bright polariton,” the light exhibits a dramatic photon antibunching effect. Remarkably, the Fano-resonant asymmetric three-photon transmission caused by the interference between the dressed states leads to a new quantum feature that is strongly nonclassical (the third-order correlation function *g*^(3)^(0, 0) ≪ 1) and has a wide and tunable bandwidth. The dependence of the intrinsic third-order correlation and time symmetry of the photon stream on the controlled parameters is also examined. Strongly nonclassical, all-optically controllable multi-photon dynamics are very important for future quantum devices and metrology.

The realization and manipulation of strongly coherent interactions between individual photons and matter will enable applications in many fields of physics ranging from solid-state^1^, atomic and laser physics^2^, to quantum information processing^3^,^4^ and to studies of strongly correlated many-body systems^5^. Cavity quantum electrodynamics (CQED) plays a central role in such systems. The well-known “vacuum Rabi splitting” (VRS) induced by the coherent exchange of energy between a single photon mode and an exciton has been extensively studied^6^,^7^,^8^,^9^,^10^. Photon blockade due to the anharmonicity of the Jaynes-Cummings (JC) ladder of eigenstates has been experimentally implemented in the field^11^,^12^. Quantum nonlinearity and the climbing of the higher manifolds of eigenstates have been observed at the large cavity driving^13^.

Recently, the third-order correlation function *g*^(3)^(*τ*_1_, *τ*_2_) has been measured to study the quantum dynamics of a strongly driven atom-cavity system^14^. Compared with the second-order correlation function *g*^(2)^(*τ*), *g*^(3)^(*τ*_1_, *τ*_2_) is a more sensitive and robust tool for observing non-classicalities in the measured photon statistics. It can provide deeper physical insight into the evolution of photon numbers and photon pairs. When a three-level atom is placed in the cavity, apart from the dressed VRS (“bright polariton,” BP), a third polariton branch appears, which has been dubbed the “dark-state polariton” (DSP) and is closely related to the phenomenon of electromagnetically induced transparency (EIT). It may be possible to realize a photon blockade with negligible losses due to large nonlinearities by quantum interference in the cavity EIT (CEIT)^15^,^16^,^17^. Very recently, the control of quantum fluctuations by studying *g*^(2)^(*τ*) in the CEIT and optical frequency combs via quantum interference with a microcavity has been proposed^18^,^19^. Although the coherent controllability in such systems holds great potential in applications of quantum engineering and quantum logic gates, these beautiful quantum phenomena still await observation.

In this paper, we investigate the third-order photon correlations of transmitted light of a Λ-type three-level atom inside a strongly coupling optical ring cavity. In contrast to *g*^(2)^(*τ*), where the dynamics are dominated by the coherent internal dynamics (VRS) of the first manifold of the dressed states, the probability for conditionally detecting a photon pair 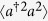 is determined by the quantum Rabi oscillations. We show that such a CEIT provides an impressive degree of optical control of the photon statistics and an intrinsic correlation at the polariton resonances in the strongly interacting systems. Super-Poissonian, Poissonian and sub-Poissonian photon statistics can be all-optically changed by an external coupling field. At the DSP, the large dispersion near the CEIT resonance leads to the cavity transmission of a coherent field (*g*^(3)^(*τ*, 0) = 1), in contrast to the strong photon-bunching behavior in a typical CQED system. When the frequency of the probe light is tuned to the BP, the photons always show antibunching. Remarkably, due to the Fano-resonant cavity transmission of three photons, a new quantum feature that is strongly nonclassical with a wide and tunable bandwidth appears in the spectra, which could be significantly smaller than the counterpart at the BP resonance. Controllable time symmetric or asymmetric photon transmission is also demonstrated in CEIT systems in which the detailed balance can be held or broken. Optical control of sequential photon transmission due to the consecutive emission of single photons and photon pairs has, to the best of our knowledge, not previously been investigated. Highly nonclassical, optically controllable multi-photons transmission will find many important applications in future quantum devices.

## Results

**Figure 1:**
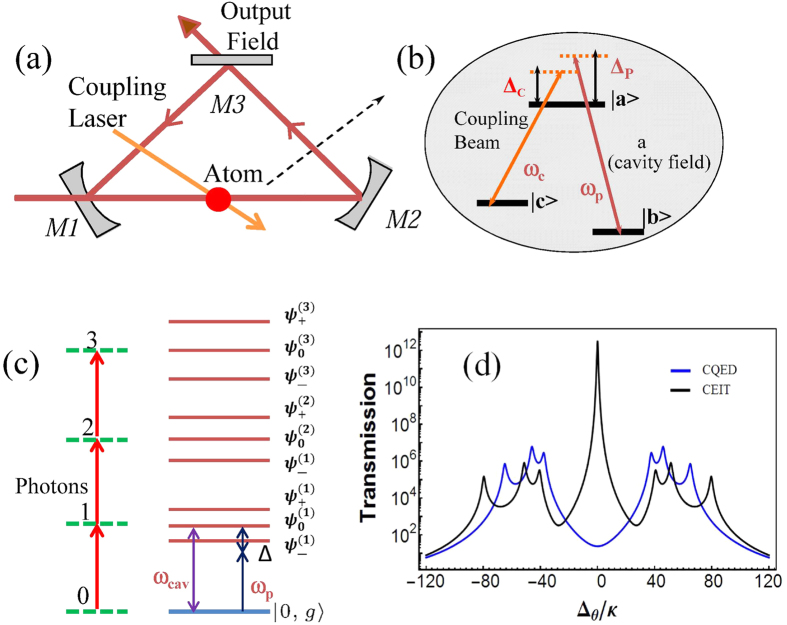
(**a**) Single Λ-type three-level atom in a weakly driven optical ring cavity. (**b**) Energy level scheme. An optical field (coupling field) of frequencies *ω*_*c*_ with Rabi frequencies Ω couples the ground state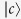 to the excited state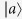; the cavity is driven by a laser field of frequency 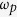 and amplitude E; the cavity field *a* couples the transition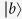 to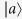 with interaction strength *g*; Δ_*p*_ and Δ_*C*_ are the single photon frequency detunings of *ω*_*p*_ and *ω*_*c*_ relative to the transition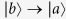 and transition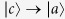, respectively. (**c**) The dressed states of the system. For each manifold there are triplet eigenstates. (**d**) The cavity transmission as a function of dimensionless cavity detuning Δ_*θ*_/*κ* for the CQED and CEIT systems.

We considered a Λ-type three-level atom in a ring cavity, as shown in Fig. 1. This system can also be realized as superconducting qubits in microwave resonators and quantum dots in microcavities. The Hamiltonian of the system in the interaction picture is given by


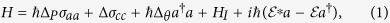


where *H*_*I*_ is the interaction term, 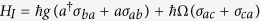. *a* represents the cavity field, which is coupled to the transition of the ground state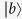 to the excited state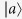. Ω is the Rabi frequency of the coupling field with frequency 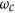 (in the EIT terminology), which drives another ground state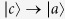 transition. *g* is the atom-cavity coupling strength. The cavity is driven by an external field with frequency *ω*_*p*_ and amplitude E. 
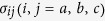
 is the operator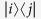 for the atom. 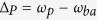 and 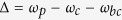 are the single photon and two-photon frequency detunings, respectively. 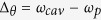 is the cavity detuning of cavity frequency *ω*_*cav*_ with *ω*_*p*_.

In the weak-cavity field limit, all of the atoms are initially prepared in the ground state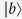, and the evolution of the system is governed by the Heisenberg equations. When the cavity field decay rate *κ*, the atomic coherence *σ*_*ba*_ decay rate *γ* and the atomic coherence *σ*_*bc*_ decay rate *γ*_*bc*_ are small, the normal modes analysis of Eq.(1) can be easily preformed. For the first manifold, the three eigenvalues are 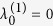 and 
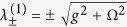
20,21. For eigenvalue zero, the normal mode is 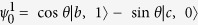, which is decoupled with the upper level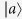, which is called the “dark-state polariton” (DSP)^22^, where 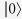, 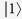 are the zero and single photon states in the cavity, respectively, and *θ* = arc tan (*g*/Ω). For the other eigenvalues 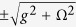, they are the VRS, *g* in CQED dressed by the coupling field, herein called the “bright polariton” (BP). We have observed the signatures of all three polariton resonances in a Doppler-broadened three-level atomic medium in the cavity^21^. Moreover, the atom-cavity polaritons can be split into two pairs of peaks at high densities or high driven laser fields because of the large nonlinearity^23^.

The three eigenvalues of the other manifolds are 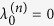 and 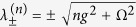. The corresponding eigenfunctions are 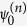, 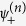 and 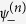, respectively. The first third-manifold is shown in Fig. 1(c). The energy difference between the adjacent triplets is 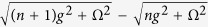 (*n* is the intracavity photon number), which is always larger than its counterpart 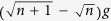 in a typical CQED system. The quantum features can be readily observed in the spectroscopic measurements of the CEIT systems, as shown in Fig. 1(d). We evaluated the probability that a photon pair will be detected at a time *τ* before or after a single photon has been observed, corresponding to the third-order correlation function^24^


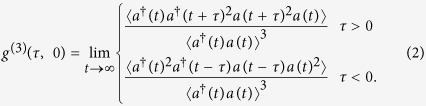


For *τ* > 0, it measures the time dependence of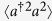 conditioned upon the detection of a single photon. For *τ* < 0, it measures the time dependence of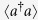, conditioned upon the detection of a photon pair.

**Figure 2:**
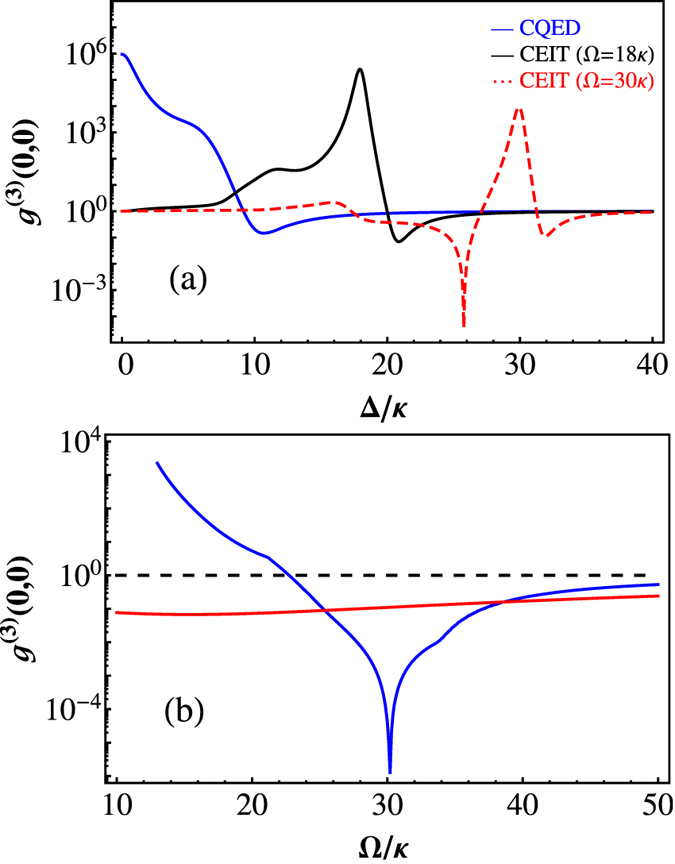
(**a**) *g*^(3)^(0, 0) as a function of the normalized two-photon frequency detuning Δ/*κ*. The blue solid line is for the typical CQED case with Ω = 0, the black curve is for the CEIT case with Ω = 18*κ*, and the red dashed curve is for the CEIT case with a large coupling strength (Ω = 30*κ*). (**b**) The dependence of *g*^(3)^(0, 0) on the dimensionless coupling field strength Ω/*κ* when the frequency of the probe is tuned to the BP (red solid curve) and at the antibunching minimum (blue solid curve) from quantum coherence. The dashed line represents the coherent transmission. The other parameters are *ω*_*cav*_ = *ω*_*ba*_, Δ = Δ_*P*_, *γ* = *κ*, *γ*_*bc*_ = 0.01*κ* and *g* = 10*κ*.

Based on the quantum regression theorem, the third-order correlation function *g*^(3)^(*τ*, 0) can be analytically derived under weak cavity driving. The probability of simultaneously detecting three photons *g*^(3)^(0, 0) as a function of the normalized two-photon frequency detuning Δ/*κ* is shown in Fig. 2. The blue solid curve represents the CQED case, i.e., Ω = 0, which shows a large bunching effect at Δ = 0. The black solid and red dashed curves are the third-order correlation functions of the CEIT with a small coupling field (Ω = 18*κ*) and a large coupling field (Ω = 30*κ*), respectively.

At the EIT resonances (DSP) and *g*, 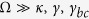, *g*^(3)^(0, 0) becomes


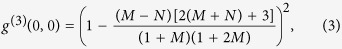


where 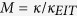, 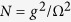, and 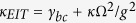 is the effective decay for the DSP. Due to the large linear dispersion near the EIT resonance, the cavity linewidth is much narrower^25^,^26^,^27^. Because 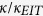 is always less than *g*^2^/Ω^2^ (i.e., *M* < *N* in Eq. (3).), it is impossible to observe photon antibunching for the DSP. For a small decay *γ*_*bc*_ or large Ω, *g*^(3)^(0, 0) ≈ 1, as shown in Fig. 2(a). The nonclassical effect of the transmitted photons appears at the BP 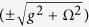 due to the anharmonicity of the triplets of the eigenstates of each manifold. For a large coupling field, the third-order correlation spectra exhibit a new quantum feature, where *g*^(3)^(0, 0) shows another impressive anti-bunching minimum, as shown by the red curve in Fig. 2(a). Its physical mechanism can be understood as follows. From Eq. (2) we know that *g*^(3)^(0, 0) is determined by the three-photon transmission, which is proportional to


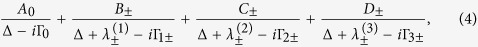


where 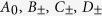 are the coupling efficiencies of the normal modes, and Γ_0_, Γ_1±_, Γ_2±_ and Γ_3±_ are the decays of the normal modes, respectively. Therefore, the three-photon transmission has either a Fano or Lorentzian lineshape because of the disparate lifetimes of the modes and their different coupling efficiencies. The amplitudes and decays of the polariton resonances in this cavity EIT system are related to Ω. For the large Ω, the widths of the modes are broadened, and the overlap of the VRS mode with the background of other modes generates a Fano-resonant asymmetric cavity transmission lineshape. The interference between the dressed states causes a new nonclassical correlation of *g*^(3)^(0, 0), with a very large tunable frequency range.

The photon statistics for these CEIT systems can be coherently manipulated by the coupling field, as shown in Fig. 2(b). For the DSP (Δ = 0), *g*^(3)^(0, 0) is always larger than one. For small Ω, CQED dominates, and the photon statistics are super-Poissonian. As Ω increases, quantum coherence takes effect, *g*^(3)^(0, 0) → 1. For the BP 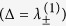, the photons statistics are always sub-Poissonian, and there is an optimized value of Ω to make *g*^(3)^(0, 0) a minimum. When the cavity detuning is close to the minimum of the Fano-like resonance, the super-Poissonian and sub-Poissonian transmission of photons can be realized by tuning only the coupling field: *g*^(3)^(0, 0) > 1 at small Ω and *g*^(3)^(0, 0) < 1 at large Ω. The anti-bunching minimum from quantum interference could cause perfect nonclassical photon transmission. With the parameters selected in Fig. 2, *g*^(3)^(0, 0) is approximately 10^−5^, which is approximately four orders of magnitude smaller than the minimum of *g*^(3)^(0, 0) when the frequency of the probe-cavity is tuned to the BP.

**Figure 3:**
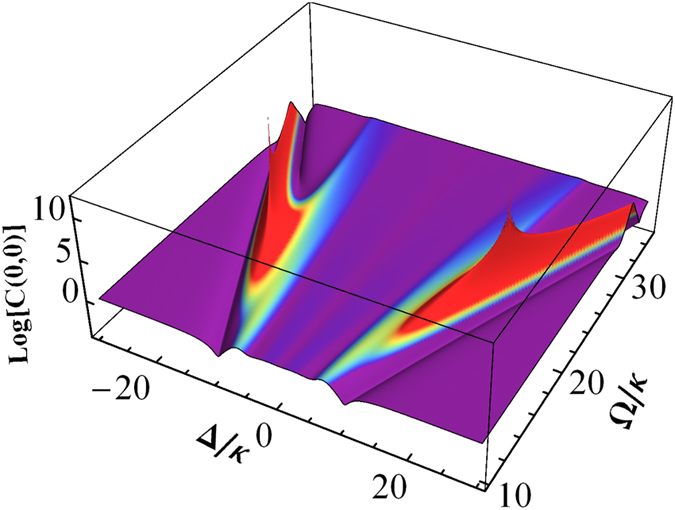
log[*C*(0, 0)] as a function of the normalized Rabi frequency Ω/*κ* and frequency detuning Δ/*κ*.

The third-order correlation function measures the conditional time evolution of the average photon number or photon pair. The quantum properties essentially come from the intrinsic photon coherence. Without the intrinsic correlations, the third-order correlation function can be completely determined by the second-correlation function 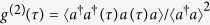. Figure 3 is a plot of 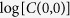 with normalized Rabi frequency Ω/*κ* and frequency detuning Δ/*κ*, where 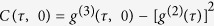
14. *C*(0, 0) is closely related to the photon statistics. If the photon statistics are Poissonian, the cavity transmission is classical, and *C*(0, 0) = 0. When the photon statistics are sub-Poissonian or super-Poissonian, the bunching and anti-bunching of the photon transmission lead to *C*(0, 0) > 0 and *C*(0, 0) < 0, respectively. It clearly shows that *C*(0, 0) is close to zero at the DSP due to the transmission of the coherent field. At the BP, *C*(0, 0) is smaller than one, and its value can be controlled by the parameters of the coupling field.

**Figure 4:**
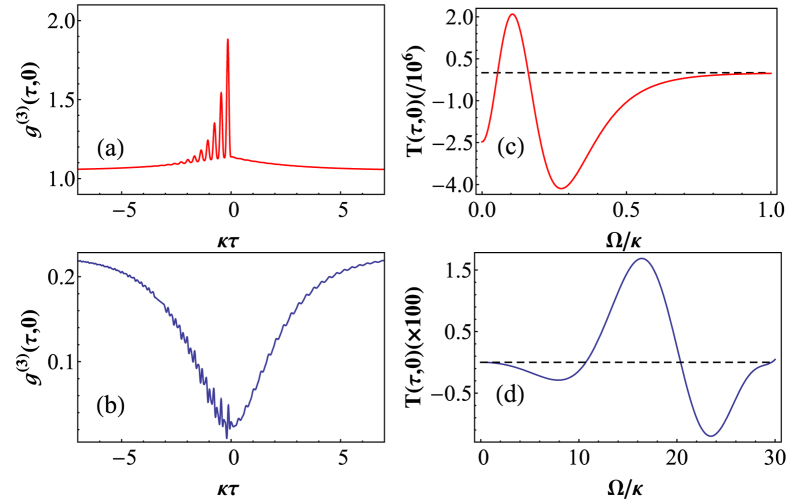
*g*^(3)^(*τ*, 0) vs. *κτ* at Ω = 15*κ* for (**a**) the DSP (Δ = 0) and (**b**) the BP 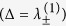. They clearly show the time-asymmetric in the evolution of three-photon correlations. *T*(*τ*, 0) vs. the Rabi frequency of the coupling field Ω for (**c**) the DSP (Δ = 0) and (**d**) the BP 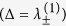. *κτ* = 1 in (**c**), and *κτ* = 0.2 in (**d**). The dashed line is for the time-symmetric transmission of photons, *g*^(3)^(*τ*, 0) = *g*^(3)^(−*τ*, 0). The remaining parameters are the same as in Fig. 2.

Finally, we investigated the time symmetry of the photon transmission in the CEIT system. Typically the third-order correlation shows the time-asymmetries, 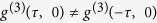, because of the different evolutions of the photon number and photon pair as well as the interference of the normal modes. Such time-asymmetric fluctuations in the output fields are a consequence of the breakdown of the detailed balance in a system driven far from thermal equilibrium^14^,^28^. The nature of the time symmetry in the CEIT system depends on the parameters of the external coupling field. Figure 4(a,b) are the plots of *g*^(3)^(*τ*, 0) as a function of *κτ* at Ω = 15*κ*. The evolution of *g*^(3)^(*τ*, 0) is determined by whether a single photon or a photon pair is detected initially, therefore the photon transmission exhibits a specific time order at the DSP and BP. Figure 4(c,d) show the difference between *g*^(3)^(*τ*, 0) and *g*^(3)^(−*τ*, 0), *T*(*τ*, 0) = *g*^(3)^(*τ*, 0) − *g*^(3)^(−*τ*, 0), with Ω for different probe-cavity detunings Δ/*κ*. When Δ is tuned to the DSP, the transmitted photon stream is asymmetric for small Ω. The third-photon correlation becomes time-symmetric at large Ω, for which there are no intrinsic correlations, and therefore *g*^(3)^(*τ*, 0) = *g*^(3)^(−*τ*, 0). In the case of the BP, 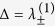, and it can be manipulated by the coupling field. The photon transmission could be time-asymmetric or time-symmetric, depending on the specific values of Ω and the cavity detuning Δ, as shown in Fig. 4(d).

We finally discuss the experimental measurement of the highly controllable photons correlations. The EIT with single atoms in a cavity has been observed in the recent experiments^29^,^30^. On the other hand, the third-order photon correlation *g*^(3)^(*τ*,0) has also been measured in the CQED^14^ and quantum dot system^31^, respectively. Although the detection of such nonclassical features would benefit from the truly strongly interaction of photons and atom, the intrinsic coherence of the cavity polaritons could greatly relax the constraints. Therefore the currently available technology especially with a smaller cavity or with better control of the atomic localization could bring our proposal into reality.

## Discussion

We have studied three-photon correlated spectra in an intracavity EIT atomic system. Higher-order photon correlations, differed more significantly than the values of *g*^(2)^(0), reveal the intrinsic quantum features of the system. The dynamical evolution of transmitted photons and photon pairs could be optically tuned by the external coupling laser. Such high-order correlations could be significant in future quantum engineering. In addition to its obvious relevance to conditional quantum dynamics^14^ and quantum-information processing, the system could be easily extended from a single photon blockade to a photon pair blockade^31^. The strikingly nonclassical effect of the quantum coherence in such higher-order photon states could be used an alternative scheme for generating the multiphoton Fock state^32^, which is important for high-resolution imaging, lithography and metrology. This all-optically controllable transmission of photons and photon pairs can be generalized to the case of multi-photon transmission with strong pumping, allowing the implementation of new generations of coherently controllable light sources such as N-photon guns or emitters of N-photon bundles^33^. Finally, in the fundamental sciences, the manipulation of photon transmission with giant nonlinearities in the EIT system also plays a central role in recent fascinating proposals for strongly interacting photon gases and many-body phenomena^34^.

## Methods

The evolutionary dynamics of the system are governed by the Heisenberg equations. The expectations of the operators are given by


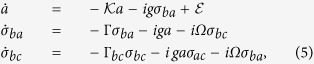


where 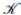 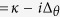, 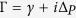 and 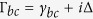. *κ*, *γ* and *γ*_*bc*_ are the decay rates for the cavity field and the atomic coherences *σ*_*ba*_ and *σ*_*bc*_, respectively.

To calculate 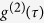 and 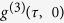, the wavefunction of the system is truncated to including the three-photon states. The expectations of the operators, including the two- and three-quanta processes, are easily derived from the Hamiltonian of Eq. (1). Using quantum regression theorem, the second-order and third-order correlation functions can be calculated.

## Additional Information

**How to cite this article**: Zhang, X. *et al.* All-optical control of three-photon spectra and time asymmetry in a strongly coupled cavity polariton system. *Sci. Rep.* **6**, 22560; doi: 10.1038/srep22560 (2016).
